# Metacognitive Cues, Working Memory, and Math Anxiety: The Regulated Attention in Mathematical Problem Solving (RAMPS) Framework

**DOI:** 10.3390/jintelligence11060117

**Published:** 2023-06-11

**Authors:** Daniel A. Scheibe, Christopher A. Was, John Dunlosky, Clarissa A. Thompson

**Affiliations:** The Psychological Sciences, Kent State University, Kent, OH 44240, USActhomp77@kent.edu (C.A.T.)

**Keywords:** metacognition, working memory, math anxiety, mathematical problem solving, state math anxiety, metacognitive experiences

## Abstract

Mathematical problem solving is a process involving metacognitive (e.g., judging progress), cognitive (e.g., working memory), and affective (e.g., math anxiety) factors. Recent research encourages researchers who study math cognition to consider the role that the interaction between metacognition and math anxiety plays in mathematical problem solving. Problem solvers can make many metacognitive judgments during a math problem, ranging from global judgments such as, “Do I care to solve this problem?” to minor cue-based judgments such as, “Is my current strategy successful in making progress toward the correct solution?” Metacognitive monitoring can hinder accurate mathematical problem solving when the monitoring is task-irrelevant; however, task-relevant metacognitive experiences can lead to helpful control decisions in mathematical problem solving such as checking work, considering plausibility of an answer, and considering alternate strategies. Worry and negative thoughts (i.e., math anxiety) can both interfere with the accuracy of metacognitive experiences as cues in mathematical problem solving and lead to avoidance of metacognitive control decisions that could otherwise improve performance. The current paper briefly reviews and incorporates prior literature with current qualitative reports (*n* = 673) to establish a novel framework of regulated attention in mathematical problem solving (RAMPS).

## 1. Introduction 

Why do some people seem to effortlessly solve math problems while other people regularly run into mental roadblocks that keep them from producing solutions? Most adults (approximately 60% of American adults reported by Handel 2016) report reasoning with rational numbers in their daily jobs. Beyond the workplace, people of all ages use mathematical reasoning to complete everyday tasks such as tipping at a restaurant, evaluating medical information, playing games, understanding sports statistics, and making financial decisions. Incorporating quantitative information in decision-making is foundational to daily life (Peters 2020), numerosity is one of the most basic dimensions upon which humans perceive the world (Dehaene 2011), and solving mathematical problems is central to learning math (Lester and Cai 2016; Passolunghi et al. 2019). The term “mathematical problem solving” represents a variety of similar, yet different, stimuli. Here, we operationalize mathematical problem solving as any multi-step task that involves the use and manipulation of numerical information. Given the prevalence of mathematical problem solving in daily life and in educational contexts, understanding individual differences that affect mathematical problem solving is of critical importance. 

The current paper explores how individual differences, such as metacognitive experiences, working memory (WM), and math anxiety (MA), are related to one another and may predict success in mathematical problem solving. We discuss the online (i.e., in the moment) cognitive (including WM) and metacognitive processes that are necessary for mathematical problem solving, and how these processes are affected by MA. Then, we introduce a novel framework of regulated attention in mathematical problem solving (RAMPS), focusing on the role of online metacognitive experiences to clarify the previously proposed WM–MA relation (e.g., Ashcraft and Kirk 2001). Next, we present qualitative, open-ended responses from two studies (*n* = 673) to elucidate the RAMPS framework mechanism of in-the-moment mathematics anxiety (i.e., state MA). In the latter half of the paper, we zoom in on the specific online relations between metacognitive experiences, MA, and WM in a five-phase approach. We conclude by discussing how themes that emerged in our qualitative data might inform future research and interventions.

**Figure 1:**
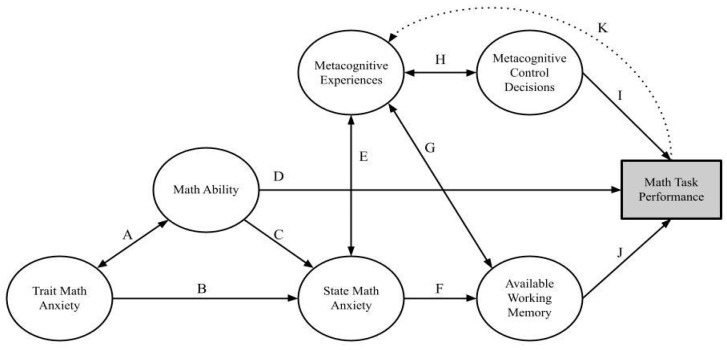
Regulated attention in mathematical problem solving (RAMPS) framework. Note: The primary use of the RAMPS framework is a reference tool to discuss the proposed interrelations between metacognitive experiences, MA, and WM during a math task. There are multiple recursive loops within this framework; thus, it is better suited as a framework for future discussions and testable models than as a testable path model.

Mathematical problem solving, broadly construed, involves cognitive (e.g., WM; Peng et al. 2016), metacognitive (e.g., feeling of error; Ackerman and Thompson 2017), and affective (e.g., MA, Hembree 1990) components. MA is closely linked to WM in the math cognition literature because it has been proposed that MA works by disrupting WM resources when one is attempting to solve math problems (i.e., disruption account). Thus, a discussion of the link between WM and metacognition in the domain of math would be incomplete without including MA. Özcan and Gümüş (2019, p. 122) previously proposed a model in which mathematical problem solving was predicted by metacognition, self-efficacy, motivation, and anxiety; yet, their model did not involve the role of WM, and metacognitive experiences were operationalized only as retrospective judgments (i.e., metacognitive judgments made after completing a task, Dunlosky and Metcalfe 2009; Rhodes 2019). The RAMPS framework builds on prior research to incorporate the role of WM and operationalize in-the-moment metacognitive experiences. See Figure 1 for an illustration of the proposed RAMPS framework. 

## 2. The Role of Working Memory in Mathematical Problem Solving 

Individuals vary in their mathematical resources and abilities; thus, what may be an intensive tax on WM via a multi-step mathematical task for one person (i.e., mathematical problem solving) may be a matter of simple recall for another person (Schoenfeld [1985] 2016). Just as a chess expert’s recall of a correct move based on prior experience with that exact situation (Schneider et al. 1993) would not be considered problem solving, a math expert in a given domain will not be said to be problem solving if the answer constitutes a recalled answer instead of a process. Evidence of expertise is demonstrated by automatic responses (recalling from long-term memory that 3 × 3 is 9) replacing algorithmic responses (using WM resources to count 3 plus another 3 plus another 3). 

Math problems vary in a wide variety of factors such as context, notation, and level of difficulty. For example, fraction addition problems (e.g., 1/2 + 1/9 = ?), math word problems (e.g., a bat and a ball cost $1.10 and the bat costs one dollar more than the ball, how much does the ball cost?), and geometric proofs (e.g., prove that two circles are concentric) are just three examples of the wide array of potential types of math that are considered mathematical problem solving for the current paper. Research in math cognition suggests that people have different affective reactions to different number types as well. For example, both adults (Mielicki et al. 2022; Scheibe et al. 2023; Sidney et al. 2021) and children (Sidney et al. 2021) report disliking fractions significantly more than other number types. It is certainly possible that differences exist in the antecedents for predicting mathematical problem solving in one math sub-domain (e.g., fraction addition) than another domain (e.g., geometric proofs).

Considering different types of math is particularly important because some forms of math rely more heavily on WM resources than others. A recent meta-analysis of WM and mathematics reported a medium correlation between the two constructs (r = 0.35; Peng et al. 2016). Many forms of mathematical problem solving involve maintaining and manipulating information to find a solution, similar to the attention-control theory of WM (Engle 2002; see also Burgoyne and Engle 2020; Cowan 2017). The attention-control theory of WM (Engle 2002) conceptualizes WM not as a number of items that can be recalled, but the ability to inhibit task-irrelevant information and focus on task-relevant information. Thus, differences in attention-control (sometimes termed the central executive; Baddeley 2001; Baddeley and Hitch 1974) are largely responsible for correlations between typical tests of WM capacity and other higher-order cognitive functions (Burgoyne and Engle 2020). 

Directed attention toward goals and subgoals is crucial to mathematical problem solving. Note that mathematical problem solving goes beyond mathematics computation in that it is a dynamic interaction between computational skills, reasoning, and metacognitive regulation. An arithmetic computation such as 2 + 2 likely may not involve the use of WM resources in adults, but mathematical problem solving that incorporates reasoning, relevant information from memory, and metacognitive regulation is a process that requires WM resources. Thus, it is unsurprising that from an individual-differences perspective, mathematical problem solving ability is linked with WM (Ashcraft 2019; Chen and Bailey 2021; Peng et al. 2016; Widaman et al. 1989). 

## 3. Processes Involved in Mathematical Problem Solving 

Cognitive processes involve the acquisition, storage, transformation, and use of knowledge (Matlin 2013). In mathematical problem solving, cognitive processes can be defined as the active processing and manipulation of stimuli. The RAMPS framework (see Figure 1) considers WM to be a cognitive process and math ability to be a composite of skills based on factors such as prior knowledge, magnitude processing (Dehaene 2011), and numeracy (Peters 2020). Beyond cognitive processes and math ability, several factors affect mathematical problem solving (Schoenfeld [1985] 2016). Problem solvers incorporate metacognitive judgments (Ackerman and Thompson 2017; Efklides 2006; Nelson and Narens 1990) and come into math environments with a rich history of attitudes toward math (Mielicki et al. 2022; Sidney et al. 2021) and affective reactions, such as anxiety prior to and during math tasks (Ashcraft 2019; Dowker et al. 2016; Hembree 1990). Mathematical problem solving never exists in a vacuum. Relations among constructs displayed in Figure 1 are discussed in subsequent sections. 

### 3.1. Metacognition and Mathematical Problem Solving

Metacognition—thoughts about one’s own thoughts and cognitions (Flavell 1979)—is studied in a variety of ways and affects many facets of everyday life (Dunlosky and Metcalfe 2009; Rhodes 2019). The RAMPS framework builds on previous work on general metacognitive frameworks (e.g., Nelson and Narens 1990) and metacognitive frameworks in meta-reasoning (Ackerman and Thompson 2015, 2017; Efklides 2006). Meta-reasoning is operationally defined as monitoring and control of reasoning and problem solving (Ackerman and Thompson 2017). This definition is similar to the current definition of mathematical problem solving; thus, models of meta-reasoning are ideal starting points from which to create a framework of metacognition in mathematical problem solving. Ackerman and Thompson (2015, 2017) proposed a model of meta-reasoning based on Nelson and Narens’ (1990) seminal framework of metacognition in learning and memory as well as Ackerman’s Diminishing Criterion Model (Ackerman 2014). Each of these models describe metacognition as a two-facet construct involving both monitoring (i.e., self-assessments) and control (i.e., actions). Metacognition in math encompasses both monitoring (e.g., “Is this solution correct?”) and control (e.g., making the deliberate choice to check one’s work) dimensions. 

Ackerman and Thompson’s (2015, 2017) model of meta-reasoning included a series of metacognitive judgments during problem solving. These judgments (e.g., initial judgment of solvability) map closely onto mathematical problem-solving processes due to the close overlap between mathematical problem solving and domain-general problem solving. In addition to judgments, problem solvers also experience less explicit metacognitive reactions, termed “metacognitive feelings” (Efklides 2006). Metacognitive feelings are elicited by nonconscious analytical processes (Efklides 2006; Koriat and Levy-Sadot 1999). These feelings and emotions (i.e., affect produced while problem solving) provide people with clues—some of which may be misleading—about the progress of cognitive processes during a task (Efklides 2006). According to Efklides (2006), metacognitive feelings interact with metacognitive judgments (i.e., judgments of learning, Dunlosky and Nelson 1992), to provide people with a continuously updating sense of their likelihood to reach a satisfying solution to the problem. 

Metacognition is central to mathematical problem solving because online metacognitive experiences or “concurrent metacognition”—specific online metacognitive feelings and judgments that interact with WM (Bellon et al. 2019; Efklides 2006; Hertzog and Dixon 1994)—occur continuously during problem solving. We argue that these metacognitive experiences interact with MA and WM to affect control decisions such as checking one’s work or altering one’s strategy. Such control decisions directly affect performance in math tasks. Additionally, retrospective metacognitive judgments may affect these same factors and interact with them to predict future iterations of mathematical problem solving (see Path K in Figure 1). 

Note that both explicit metacognitive judgments and implicit metacognitive feelings are encompassed in “metacognitive experiences”. Metacognitive feelings represent an important component of the RAMPS framework because solving math problems is often an emotionally charged experience (Ashcraft 2002; Dowker et al. 2016). Although problem solvers may not often make explicit judgments about their emotional state (e.g., “What level of math anxiety am I experiencing at this moment?”), feelings and emotions clearly run concurrently with the cognitive processing of mathematical stimuli. The variety of metacognitive experiences illustrates the potential for investigating many open questions in the domain of mathematical problem solving. 

### 3.2. Math Anxiety and Mathematical Problem Solving

Metacognitive experiences (i.e., judgments and feelings) occur continuously during mathematical problem solving. These metacognitive experiences not only affect control decisions (e.g., checking one’s work or giving up), but they can also dictate changes in affect. Carver (2003) and Carver and Scheier (1998) proposed a two-loop feedback model of affect in problem solving that highlights how positive affect can broaden the scope of attention. People incorporate metacognitive experiences, whether consciously or unconsciously, that affect their online control decisions. For example if a person notices that they are struggling with a complicated problem, they might work harder through an approach process (see Carver and Scheier 1998). However, a math anxious individual will likely be more prone to avoidance and would be likely to spend less time attempting to complete the problem than they otherwise might have in the absence of a negative affective reaction, especially if the individual is metacognitively aware of negative affect. People often become anxious while solving math problems (Ashcraft 2002), so much so that MA is often likened to a specific phobia (Ashcraft and Ridley 2005). 

## 4. Working Memory and Math Anxiety

A consistent, moderate relation between math performance and MA is regularly cited in math cognition literature (Barroso et al. 2021; Caviola et al. 2022; Hembree 1990; Ma 1999; Namkung et al. 2019; Zhang et al. 2019). Seminal research on MA (e.g., Dreger and Aiken 1957; Richardson and Suinn 1972) treated MA as a stable personality construct. Similarly, in the RAMPS framework, we consider trait MA to be a personality construct. However, MA is also a cognitive construct (Ashcraft 2019) in that worry or fear during a math task is an internal process that disrupts the cognitive system while problem solving (Eysenck 1992; Eysenck and Calvo 1992). The most common construct posited to mediate the math performance–MA relation is WM (Pellizzoni et al. 2021), because MA during a math task is posited to disrupt WM resources (Ashcraft and Kirk 2001). Thus, little debate remains in the literature that both (a) WM is important in mathematical problem solving and (b) WM interacts with MA in some way to predict math outcomes. However, many open questions remain regarding this interaction. One open question is how metacognition—specifically online metacognitive experiences—affects the WM–MA interaction. A proposed framework is presented in Figure 1. 

Of course, further research will be required on MA and WM to completely operationalize both constructs. Often in WM research, the term “working memory” is used without a clear definition (Cowan 2017). Yet, as Cowan points out, at least nine different definitions of WM and short-term storage currently are used in the WM literature (Cowan 2017, p. 1159). Perhaps a main reason that the mechanism by which MA exerts its influence is yet to be fully understood is because of the vast variability in the operationalization of related constructs, such as WM. WM in math cognition is often referred to as both a system and a capacity or resource (e.g., Beilock and Carr 2005; Justicia-Galiano et al. 2017; Ng and Lee 2019; Passolunghi et al. 2019; Peng et al. 2016; Ramirez et al. 2013). From our perspective, it may be easiest to consider WM from an attention-control model (e.g., Engle 2002; Unsworth and Engle 2007) or a multicomponent system (e.g., Baddeley 2001; Baddeley and Hitch 1974) for the purposes of considering the MA–math performance relation (see the Discussion section for an extended argument and recommendations for researchers). WM is often referred to as a processing resource or capacity in the MA literature (e.g., Passolunghi et al. 2019); thus, we adopt an attention-control perspective on WM (e.g., Engle 2002). 

### 4.1. The Mechanism of State Math Anxiety

Our primary focus is on the relation between WM and metacognitive experiences in mathematical problem solving; however, because MA interacts with WM to predict mathematical problem-solving accuracy, elucidating the mechanism of MA is relevant to the current argument. Discussions of interventions specifically for MA are outside the scope of the current paper (but see Barroso et al. 2021; Dowker et al. 2016; Ganley et al. 2021; Mammarella et al. 2019; Ramirez et al. 2018; Scheibe et al. 2023), but clarifying the causes of state MA can help explicate the relation between WM and metacognitive experiences in mathematical problem solving. We focus on WM (as opposed to other executive functions, Miyake et al. 2000) because WM is the postulated mechanism in the disruption account of math anxiety; thus, WM is a central component of the RAMPS framework. 

#### 4.1.1. The Disruption Account of Math Anxiety

At least three theoretical models of MA currently exist in the math cognition literature. The most highly cited model of MA (Ramirez et al. 2018) is the “disruption account” championed by Ashcraft and colleagues (Ashcraft 2002; Ashcraft and Faust 1994; Ashcraft and Kirk 2001; Ashcraft and Krause 2007; Faust et al. 1996). This account treats MA as a cognitive construct and builds on prior work outside the domain of math: the processing efficiency theory (Eysenck 1992; Eysenck and Calvo 1992). The primary tenets are that cognitive worry is an internalized process that consumes cognitive resources during an anxious reaction (Ashcraft 2019). Importantly, Ashcraft (2019) notes that MA can be disruptive at a dual-task level (e.g., cognitive worry creating task-irrelevant thoughts) or at a metacognitive level (e.g., failure to inhibit attention to worries, also creating task-irrelevant thoughts). Note that prior work in math cognition does not clearly label the latter negative effect of MA as metacognitive, but by the most parsimonious definition of metacognition (i.e., thinking about thinking; Flavell 1979), directing attention to cognitive worries is inherently metacognitive. We extend this prior work to explicitly address the differences between cognitive worry creating a dual-task paradigm and meta-level task-irrelevant cognitions caused by anxious reactions (see Section 4.2.2 on Phase 2: Progress Evaluations). 

Because WM is a processing resource, any moderation of WM on the MA–math performance relation would be expected to be in-the-moment (i.e., “state”) effects. Thus, because the disruption account proposes decreased math task performance due to increased MA through decreased WM resources (see Figure 1), this account would predict state WM to largely, if not entirely, account for the MA–math performance relation (although see Ashcraft 2019 for a discussion of MA as a multifaceted phenomenon). Math cognition researchers disagree regarding the nature of the MA–math performance link in terms of causal direction (Ashcraft 2019; Carey et al. 2016; Dowker et al. 2016; Mammarella et al. 2019; Ramirez et al. 2018). For example, one explanation is that MA causes a decrease in math performance due to its in-the-moment effects on mathematical problem solving (the disruption account; Ashcraft and Kirk 2001). Another explanation is that when people are not good at math, that deficit causes MA (the deficit account; Maloney 2016). A third explanation is that the MA–math performance link is driven by how one interprets math situations (the interpretation account; Ramirez et al. 2018). 

We address the disruption account’s state effects of MA on math performance through WM; however, other accounts (e.g., the deficit approach and the interpretation account; see Ashcraft 2019; Ramirez et al. 2018 for reviews) may be better suited to explain how math experiences inform trait MA. Such relations (e.g., math self-concept predicts MA; Ahmed et al. 2012) are important in influencing trait MA and trait math ability but are outside the scope of the current paper and thus are not modeled in Figure 1. Instead, we incorporate qualitative data and a novel framework to argue for how competing theories of MA might exist more harmoniously. 

#### 4.1.2. Factors Inducing State Math Anxiety

Where do online MA feelings originate? Scheibe et al. (2023)^1^ collected two samples of open-ended responses about MA experiences from college students (total *n* = 673 independent participants). The primary aim of Scheibe et al. (2023) was to assess the efficacy of different MA interventions (e.g., expressive writing). However, as part of those two studies, participants answered several open-ended questions about MA, such as: “What types of situations make you feel the most anxious about math and why?” Open-ended responses to these questions were analyzed and coded for several different potential causes of MA. As shown in Table 1, 46.1% of students reported that testing situations or high stakes situations induced anxiety, 30.5% reported that social pressure or fear of embarrassment induced anxiety, and 20.3% reported that specific number types induced anxiety. 

These qualitative data provide a data-driven perspective on what factors induce anxiety during math situations. Participants’ responses also provide insights into the interrelations displayed in Figure 1. For example, one of the primary reasons participants reported MA is that they were fearful of social judgment, i.e., of embarrassment due to peer evaluation. It may be much easier to identify as “not a math person” than to put forth one’s best effort on mathematical problem solving in social situations, make an error, and “look like an idiot,” as one participant described it. Thus, it appears that one primary way that MA might be alleviated in the future is to foster learning environments, both formal and informal, that allow learners to be incorrect. Fear of failure appears to be a primary motivator for MA, which often leads to math avoidance (Erickson and Heit 2015; Morsanyi et al. 2016). Consider for example, one participant’s anecdote:

“For me, it’s being called on by a teacher. Just remembering this now, I remember one day in elementary I had this one teacher who called on me to answer a simple fraction problem and I didn’t know the answer to it. The teacher became frustrated at this, and she kept demanding the right answer. Every single time, I would guess and get the answer wrong, eventually to the point where she started yelling at me and I started crying. I think from this point on, I just avoided being picked on, even if I knew the answer, it really took a toll on my confidence towards math.”

Note the closing sentence of this anecdote. This participant is demonstrating clear metacognitive control to avoid math situations due to anxiety based on prior situations. This is just one example of how intrusive thoughts regarding fear of judgment and ensuing embarrassment can either (a) disrupt online WM or (b) cause the problem solver to avoid putting in effort on the problem altogether. 

One way that the induction of MA can affect the WM-metacognitive experiences relation is that online feelings of MA (i.e., state MA) appear to often be driven by metacognitive judgments. For example, in line with prior research on time-limited testing and anxiety (Boaler 2014; Devine et al. 2012; Kellogg et al. 1999), qualitative evidence suggests that one main cause of MA might be time constraints during mathematical problem solving (see Table 1). In order for problem solvers to feel state MA due to time constraints, they must make some judgment comparing how much time they have to complete the math task and how much time they require to complete the task under current conditions. To illustrate this point, consider two different time constraints. Both scenarios involve solving 20 fraction addition problems. In scenario A, the time constraint is three hours. In scenario B, the time constraint is 20 min. In order for the time constraint to be relevant to the problem solver in scenario A, their average time to complete one fraction addition problem must be 6 min or more. However, to complete all problems in scenario B, the problem solver must complete one problem per minute. Thus, it could be predicted that whether MA due to time constraints is experienced should be a function of the problem solver’s evaluation regarding if they have adequate time. Following this initial assessment, the astute problem solver will likely re-evaluate their initial assessment based on their progress. For example, in scenario A, if the problem solver completes the first fraction addition problem in 60 s and is metacognitively aware that they are well ahead of the schedule they must maintain to complete all problems on time, they should dismiss the time constraint as a factor, or at least re-assess at a later point. Note, however, that if the problem solver in scenario B completes the first problem in 60 s, that would likely induce anxiety due to being exactly on pace, with little room for error. Thus, metacognitive judgments affect MA both at the beginning of, and throughout mathematical problem solving. 

### 4.2. Phase Approach to Relations between Working Memory and Metacognitive Experiences

**Figure 2:**
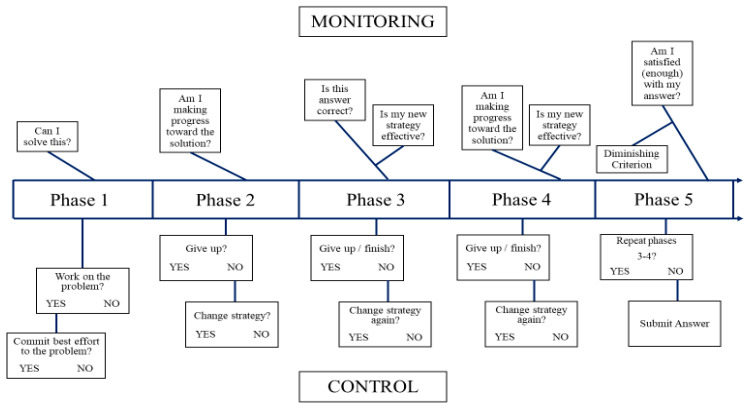
5-Phase Framework of Monitoring and Control in Mathematical Problem Solving.

Nelson and Narens’ (1990) seminal framework of metacognition posited that metacognition is a series of evaluations (monitoring) and decisions (control) that connect the meta level and the object level (Ackerman and Thompson 2015). We applied this framework to mathematical problem solving with special attention to metacognitive experiences and the relation between MA and WM (see Figure 1). From an attention-control perspective of WM (e.g., Engle 2002; Burgoyne and Engle 2020), WM resources are necessary for both working through the math problem (object level) and the maintenance and updating of progress (meta level). Metacognitive experiences cannot simply be broken into one construct in a path model (e.g., Figure 1) because these experiences vary in several aspects including time (i.e., predictive, concurrent, and postdictive) and type of processing (i.e., explicit or implicit). Thus, we propose a path model (see Figure 1) that can be revised and tested, but we also propose a 5-phase framework (see Figure 2) based on monitoring and control processes (Nelson and Narens 1990). By combining the big picture path approach and the microanalysis of the 5-phase approach, we present a wide range of open empirical questions.

One facet of metacognition, metacognitive monitoring, is a crucial component in mathematical problem solving because online metacognitive judgments inform and predict whether a person will initiate, terminate, or change effort in a cognitive task (i.e., metacognitive control; Ackerman and Thompson 2017). Metacognition is often studied by examining judgments given after the problem solving (cf. Özcan and Gümüş 2019). These retrospective judgments are often more accurately aligned with performance than are predictive judgments (Dunlosky and Metcalfe 2009; Rhodes 2019); thus, retrospective judgments are often used as a, if not *the*, measure of metacognition in empirical studies (Özcan and Gümüş 2019). Retrospective judgments are displayed in Figure 1 by Path K, but note that Path K does not encompass all possible online metacognitive experiences. A rich variety of meta-reasoning judgments and metacognitive control decisions coincide with the temporal evolution of solving a cognitive task. It is this cycling of judgments and metacognitive feedback loops (i.e., online judgments) during mathematical problem solving that the proposed framework in Figure 2 highlights. An important note about Figure 2: This figure was designed to apply Nelson and Narens’ framework to the path model displayed in Figure 1 by incorporating the data from Table 1. In other words, traits certainly affect problem solvers (e.g., paths B, C, and D in Figure 1), but Figure 2 applies a phase approach to the online relations between WM, MA, and metacognitive experiences (i.e., paths E, F, G, and J in Figure 1) that affect problem-solving performance. Each phase of Figure 2 will be broken down separately with mention of specific metacognitive experiences based on prior work (Ackerman and Thompson 2017; Efklides 2006). For an example to illustrate the phases, we present the following problem taken from the cognitive reflection test (Frederick 2005): If it takes 5 machines 5 min to make 5 widgets, how long would it take 100 machines to make 100 widgets? 

#### 4.2.1. Phase 1: Initial Evaluation

What cues do people use to evaluate a problem when they are first presented with it? Depending on the time constraints and how much problem solvers are motivated to make their best effort on a problem, a variety of explicit judgments and implicit feelings might be employed. The primary monitoring judgment displayed in Figure 2 is “Can I solve this?” This judgment can occur explicitly or implicitly—a common theme for metacognitive judgments during mathematical problem solving is that they are often automatized (thus, becoming not metacognitive by most definitions, Flavell 1979; Hacker 1998). However, whether an explicit question to the self or an implicit metacognitive feeling (Efklides 2006), the answer to this initial monitoring evaluation will dictate whether the problem solver attempts the problem. If the individual believes themself to be entirely incapable of solving the problem, what use is there to try? But how do people make this initial evaluation?

According to prior research on meta-reasoning, people make an initial judgment of solvability (Ackerman and Thompson 2017; Thompson 2009; Topolinski and Strack 2009) at the onset of a problem. Importantly, this judgment is not only that the problem is solvable (i.e., it is possible that an expert could solve the problem), but that the problem is solvable by the problem solver (i.e., it is possible that *that* person can solve the problem; Ackerman and Thompson 2017). We argue that people use many cues to inform this decision in the domain of mathematical problem solving, such as: (a) feeling of familiarity, (b) initial feeling of difficulty, and (c) math self-perceptions. Feeling of familiarity refers to the sense of previous occurrence with a stimulus (Efklides 2006; Nelson et al. 1998). Feeling of difficulty is a sense of challenge associated with a particular problem due to perceived likelihood of error, lack of available response, or the need to invest more time or effort (Efklides 2006; Efklides et al. 1999). Feeling of familiarity tends to be associated with positive affect and feeling of difficulty tends to be associated with negative affect (Efklides 2006). That is, people tend to like familiar stimuli, and dislike challenging stimuli. Finally, math self-perceptions are an aggregated individual difference specific to the domain of math that influences people’s motivation to interact with math stimuli (Lee 2009). These self-perceptions include math self-concept (Ahmed et al. 2012), math self-efficacy (Pajares and Miller 1996), and math attitudes (Mielicki et al. 2022; Sidney et al. 2021). Math self-perceptions act as a phase 1 cue. Even if the other feelings are at odds with math self-perceptions, math self-perceptions might override them. For example, consider a person who feels that the machines and widgets problem is both unfamiliar and difficult, yet they consider themself to be good at math, so they evaluate it as solvable anyway, despite the conflict between the cues. Interpretation of these feelings leads to a decision on the initial judgment of solvability, which directly affects the control decisions in phase 1. That is, an individual who judges that they are 100% capable of solving a problem is much more likely to not only attempt the problem, but to attempt it with a motivated effort. At this point, the problem solver is ready to choose a strategy and begin the problem. 

#### 4.2.2. Phase 2: Progress Evaluation

During phase 2, problem solvers attempt to start making progress toward the solution and metacognitive and affective influences contribute. The problem solver can advance toward the solution by engaging in appropriate mathematical steps (cognitive processes), ideally while evaluating the efficacy of the steps (metacognitive processes) and inhibiting task-irrelevant distractors (e.g., MA) that can interfere with WM resources necessary for solving the problem (recall that we define WM as a processing resource of limited capacity; Baddeley and Logie 1999). 

Metacognitive processes in phase 2 center on the question, “Am I making progress toward the solution?” (see Figure 2). Two facets involved in feelings of difficulty are estimates of effort and estimates of time required for problem solving (Efklides 2006; Efklides et al. 1999). Problem solvers generate feelings about these factors, whether implicit or explicit, during phase 1 while attempting to generate a judgment of solvability. Thus, by phase 2, problem solvers have an existing expectation for how long they feel the problem should be taking them to solve and how much effort it should require. These estimates during phase 1 are informed by a variety of factors specific to the problem solver (e.g., math self-efficacy; Pajares and Miller 1996), the stimulus type (e.g., fractional components versus whole number components; Mielicki et al. 2022), and environmental factors (e.g., time constraints; Scheibe et al. 2023, mood; Efklides and Petkaki 2005). All of these factors are considered, typically implicitly, and the problem solver develops feelings about appropriate effort and timing. During phase 2, these feelings and expectations are compared to the progress being made on the problem. 

Comparison to expectations can elicit positive affect (e.g., elation, eagerness, relief, or calmness; Carver and Scheier 1998) or negative affect (e.g., sadness, depression, fear, or anxiety; Carver and Scheier 1998). For example, if progress toward the solution comes quickly and easily to an individual who was expecting the problem to take a lengthy amount of time to solve, that person may experience positive affect due to being above their expected baseline in terms of effort and timing. The opposite is unfortunately also true. Underperforming against expectations of effort and timing often leads to negative affect, particularly MA. Feelings of fear or apprehension related to math stimuli (i.e., MA, Richardson and Suinn 1972) often are paired with physiological responses (Pizzie and Kraemer 2021), similar to other forms of anxiety (Dowker et al. 2016). Anxious responses include hands shaking, palms sweating, heart racing, limbs bouncing, and feeling like one’s brain is overwhelmed. These reactions are particularly important for two reasons. First, by the disruption account of MA (Ashcraft 2019; Ashcraft and Kirk 2001; Faust et al. 1996), MA depletes available WM resource by introducing task-irrelevant thoughts, thereby causing decreased math performance. We also extend the argument of the disruption account to posit that MA not only affects available WM resources, but MA itself, in the form of physiological responses, is a metacognitive cue for problem solvers. That is, state MA is a dual burden in that it directly taxes WM resources with task-irrelevant processing, but it can also be an observable cue that may lead to further distractions from the task. 

To illustrate this point, consider an individual who notices that they are struggling with the machines and widgets problem. They thought they would probably be able to solve it without much time or effort (phase 1 monitoring judgment), but now that they have begun to try and solve the problem, they do not know where to begin. After several moments of not making any progress, they notice that their leg is bouncing and their brain suddenly feels clogged. These anxious physiological responses are an activation of the autonomic nervous system, and although the math problem presents no physical danger, the problem solver has a decision to make: fight or flight. 

Control decisions based on monitoring evaluations of not making progress during phase 2 include giving up or skipping (flight) the current problem (more likely with individuals experiencing high levels of state MA; Bellon et al. 2021), or pivoting to a different strategy (fight; Berardi-Coletta et al. 1995). If instead problem solvers evaluate that they are making progress, they are likely to continue taking steps with their current strategy.

#### 4.2.3. Phase 3: Intermediate Evaluation

At phase three, problem solvers generate an initial response and must decide whether to provide that response as their answer, or continue working on the problem (see Ackerman and Thompson 2017, Figure 1, p. 611). Prior research on meta-reasoning by Ackerman, Thompson, and colleagues (Ackerman and Thompson 2017; see also Ackerman 2014; Ackerman and Beller 2017; Thompson 2009; Thompson and Johnson 2014; Thompson et al. 2011, 2013) proposed different possibilities for how problem solvers develop a final answer. Two of these possibilities are the Metacognitive Reasoning Theory (see Thompson 2009) and the Diminishing Criterion Model (see Ackerman 2014). The Diminishing Criterion Model (Ackerman 2014; Ackerman and Thompson 2017) informs the mechanism of how problem solvers reach a final answer. During phase 3, problem solvers make one or more internal evaluations about the accuracy of potential solutions (Ackerman and Thompson 2017). These evaluations are captured by judgments of intermediate confidence. According to the Diminishing Criterion Model (Ackerman 2014), as time passes during the problem-solving process, problem solvers are increasingly more likely to provide a final response that they endorse with less confidence. 

Meta-reasoning research has involved tasks that are more mathematical (e.g., cognitive reflection task; Frederick 2005) and less mathematical (e.g., remote associates test; Mednick 1962). We argue that meta-reasoning research effectively informs metacognitive research in mathematics because math is fundamentally relational in nature (Thompson et al. 2023). Thus, even though many people treat math differently than other academic subjects (Erickson and Heit 2015), and it has been argued that MA might be similar to a specific phobia (Ashcraft and Ridley 2005), the RAMPS framework is informed by several existing parallels from prior research in neighboring domains.

#### 4.2.4. Phase 4: Second Progress Evaluation

Phase 4 is a combination and extension of phase 2 and phase 3. This phase is similar to phase 2 in that it involves active problem solving with monitoring components focused on evaluations of progress. These evaluations are based on similar cues to phase 2: comparison to expectations of ease, effort, and time required, and monitoring of physiological reactions (e.g., MA). The same interactions between metacognitive experiences, MA, and WM that are present in phase 2 are also present in phase 4. These are the active problem-solving phases. Based on the metacognitive judgments and feelings in phase 4, problem solvers can continue working on the problem based on their current strategy, change strategy again, or give up. Note that phases 3 and 4 can repeat in multiple sequential loops depending on how many different strategies the problem solver attempts prior to reaching the diminishing criterion for confidence (Ackerman 2014). Eventually, a final answer is provided, which takes the problem solver to phase 5. 

#### 4.2.5. Phase 5: Final Answer Evaluation

Phase 5 is all about the final answer. Problem solvers engage in several possible solutions during problem solving that could become the final solution, but if more active problem solving or strategy switching takes place following coming up with the solution, such efforts would fall under phases 3 and 4; not phase 5. Phase 5 is the retrospective counterpart to the predictive phase 1. Just as in phase 1 problem solvers make judgments about solvability, how hard the problem might be, how prepared they are to attempt to solve the problem, how familiar they are with the problem features, etc. Problem solvers are capable of making explicit metacognitive judgments in phase 5 based on implicit judgments and feelings. Common examples of retrospective metacognitive judgments are confidence judgments (e.g., “How confident are you in your answer, from 0% = Not at all confident to 100% = Completely confident?”; Dunlosky and Metcalfe 2009; Fitzsimmons et al. 2020; Rhodes 2019; Scheibe et al. 2022). But what cues do participants use to make these judgments, and why are they important?

According to Ackerman and Thompson (2017), problem solvers make judgments including final confidence, feeling of error, and final judgment of solvability. Further metacognitive feelings include judgment of solution correctness (Efklides 2006) and feeling of satisfaction (Efklides 2002, 2006). Collectively, problem solvers have a sense of whether they committed an error, they might be right, or they are certainly right (Ackerman and Thompson 2017; Efklides 2006; Fitzsimmons et al. 2020; Gangemi et al. 2015). These feelings are not foolproof; indeed, even though retrospective judgments are better predictors of task accuracy than predictive judgments, they are rarely perfectly aligned with accuracy (Rhodes 2019). Problem-solvers’ feelings about their final solution may affect task performance and more broadly their own self-perceptions.

For example, consider an individual who has spent approximately a minute trying to solve the widgets and machines problem. That person considered multiple different strategies and attempted the problem from multiple angles, yet is still not confident with the solution they chose. A relevant task-specific effect might be that they have multiple different problems to solve and will approach the next problem differently based on their low confidence about their answer to the widgets and machine problem (see Path K in Figure 1). If multiple low-confidence judgments are made during one session, it is also possible that the individual will assimilate these judgments into their self-perceptions (e.g., “I thought I was good at math, but I did not know how to solve any of these problems, so maybe I am not as good as I thought”). Both the task-specific and downstream implications are discussed in future directions.

## 5. Conclusions and Future Directions

We proposed the RAMPS framework based on prior work on metacognition (Efklides 2006; Nelson and Narens 1990) and meta-reasoning (Ackerman and Thompson 2017). For the remainder of the paper, we discuss why the domain of math is a logical extension of meta-reasoning research, future extensions of the RAMPS framework, and how it could inform future interventions. 

### 5.1. Extending Meta-Reasoning into Mathematics

Meta-reasoning researchers have argued that the processes of thinking and reasoning might easily be described using models of memory (Thompson and Feeney 2015, p. 7). We argue that this logic can be extended to the domain of mathematics. Indeed, meta-reasoning research often overlaps with mathematical concepts (e.g., cognitive reflection; Ackerman and Thompson 2017). The close connections between metacognitive processes and meta-reasoning are likely due to an underlying factor in both: relational reasoning. Both reasoning tasks and math tasks often involve the ability to apply rules and transfer knowledge to novel domains. Thus, the RAMPS framework significantly relies on prior work in meta-reasoning to draw extensions into the domain of math.

The RAMPS framework is novel in that it incorporates a path model involving state and trait MA, as well as metacognitive experiences and WM in predicting mathematical problem solving. We also offered a five-phase framework to zoom in on the central, yet recursive, components of the RAMPS framework to describe the cues that problem solvers may use. Focusing solely on retrospective judgments may provide valuable post-hoc information about problem solving, but doing so overlooks the wealth of cues and judgments made during phases 1–4 (see Figure 2). Additionally, WM is crucial to mathematical problem solving (Ashcraft and Kirk 2001; Peng et al. 2016). The RAMPS framework adopts an attentional-control (Engle 2002) model of WM. Indeed, we refer to the focal point of our framework as regulated attention. Attention-control is just one of several WM models, however (Cowan 2017), and future research should investigate the best model(s) of WM to employ (e.g., Ng and Lee 2019) for research at the nexus of metacognition, math cognition, and cognitive science.

### 5.2. Extensions, Interventions, and Future Directions 

A primary aim is to propose clarifying relations between metacognitive experiences, WM, and MA in mathematical problem solving. Theoretical contributions to elucidate these relations are valuable; but could this work be extended to improve mathematical problem-solving outcomes? That is, could metacognitive experiences be manipulated to decrease state MA and thus relieve the task-irrelevant taxing of WM? These and many other open questions should be investigated using experimental methods to explore and test the RAMPS framework. For example, to date, light-touch MA interventions have mostly been unsuccessful (Ganley et al. 2021; Scheibe et al. 2023). It is possible that understanding metacognitive processes and developing interventions based on this understanding might be a promising new frontier in MA interventions (Morsanyi et al. 2019). Future research can manipulate the number of metacognitive experiences or draw participants’ attention to specific metacognitive experiences during problem solving to attempt to affect participants’ task performance and task interpretation. Indeed, the recently proposed interpretation account of MA (Ramirez et al. 2018) focuses not on the math situations or an individual’s mathematical ability, but meta-level interpretation of math stimuli to be the cause of anxious reactions. We posit that the RAMPS framework may help to bridge the gap and help interdisciplinary researchers understand interrelations at the nexus of metacognitive research, cognitive research, clinical research, and research specifically on math cognition. 

Future research should target specific components of the RAMPS framework. One way to do this is to test the RAMPS from a structural equation modeling approach. A challenge in conducting this type of research is that operationalizing metacognitive regulation in mathematical problem solving can be difficult (Zepeda and Nokes-Malach 2023). Researchers must develop creative designs to tap both explicit and implicit processes. Recall that what might be an explicit step-by-step process for a novice problem solver might be an implicit recall process for an expert. Thus, individual differences in mathematical problem-solving ability pose unique research challenges that future research should aim to address. Additionally, future research could delve into the five-phase approach to empirically test or manipulate the cues used in mathematical problem solving. For example, Fitzsimmons and Thompson (2022, 2023) presented participants with familiar or unfamiliar fractions in order to manipulate the cues (e.g., familiarity) participants used to determine their confidence with predicting where to place the fractions on a number line. This is one of many ways to manipulate participants’ reliance on individual cues that are used during mathematical problem solving. Similar procedures could be used to manipulate the salience or presence of cues used in mathematical problem solving to test different elements of the RAMPS framework. 

Future research could also use the RAMPS framework to investigate other open questions in math cognition, such as why women often report greater levels of MA (Devine et al. 2012) and lower levels of confidence (Rivers et al. 2020) than do men, despite having equal math abilities. It is possible that research methodologies inspired by the RAMPS framework might lead to a deeper understanding of this issue. Gender differences are just one example of a long-discussed topic in math cognition that could potentially benefit from research derived from the RAMPS framework.

### 5.3. Final Thoughts

The current paper has offered a novel framework for future research at the nexus of math cognition, WM, and metacognition. Many open questions remain in the RAMPS framework, and many empirical studies must be conducted to test the claims we have made herein. It is our hope that the current conceptualization of relations between WM, MA, and metacognitive experiences during mathematical problem solving will be provocative and facilitate future interdisciplinary work. Morsanyi et al. (2019) recently proposed that research on metacognitive processes, MA, and WM has the potential to “significantly expand the scope of metacognitive investigations and provide novel insights into individual differences in the metacognitive regulation of learning and problem solving” (Morsanyi et al. 2019, p. 147). We thoroughly endorse this view, and hope that interested readers will join us in seeking empirical answers to the open questions.

## Figures and Tables

**Table 1:** Coding of Participants’ Open-Ended Math Anxiety Responses in (Scheibe et al. 2023).

Code	Code Definition	Examples	Prevalence
Testing or High Stakes	Any mention of (a) testing situations or (b) high-stake ramifications inducing anxiety.	*“Important exams and [the] ACT because the grade matters a lot.”*	46.1%
		*“Exams. I hate tests.”*	
Social Pressure or Embarrassment	Any mention of (a) being watched, (b) being judged, or (c) being embarrassed due to social comparison inducing anxiety.	*“When people depend on me or people are watching me because I don’t want to disappoint them.”*	30.5%
		*“When I have to express my math abilities to others. It’s easy to mess up, and that would be embarrassing.”*	
Specific Type of Math	Any mention of a specific type of math inducing anxiety (as opposed to math anxiety as more of a generality).	*“Anything that requires percentages and needs to be quickly determined.”*	20.3%
		*“Fractions and word problems. I have never been good at fractions and word problems can be very confusing.”*	
Surprise or Lack of Preparation	Any mention of being put on the spot to complete math or having to do math without the chance for proper preparation inducing anxiety.	*“When I am put on the spot because I do my best work when I have time to prepare and study.”*	10.4%
		*“Pop quizzes because it is unexpected.”*	
Time Constraints	Any mention of a specific allotted amount of time inducing anxiety.	*“Anything that requires percentages and needs to be quickly determined.”*	7.7%
		*“When I have to do it in a time limit.”*	

*Note*. The codes were not mutually exclusive. That is, a participant’s answer could be coded for none of the five codes, one of the codes, or a combination of multiple codes. An example of this overlap in the coding scheme is included in the “Time Constraints” and “Specific Type of Math” examples. Authors DAS and CAT coded 25% of the data with an interrater reliability of 0.95. The authors discussed the few disagreements, and author DAS did the remaining coding based on the high initial level of agreement between coders.

## Data Availability

The qualitative data presented in Table 1 are part of a currently embargoed dataset. They will be publicly available on OSF at the time of the publication of Scheibe et al. (2023).

## Note

1 The qualitative data presented herein (see Table 1) were analyzed using codes generated separately from Scheibe et al. (2023) and have not been analyzed or published in any other outlet.
